# Implementing and scaling verbal autopsies: into the unknown

**DOI:** 10.1186/s12916-020-01527-8

**Published:** 2020-03-09

**Authors:** Ross M. Boyce, Raquel Reyes

**Affiliations:** 1 Division of Infectious Diseases, University of North Carolina at Chapel Hill, 130 Mason Farm Road, Chapel Hill, NC 27599 USA; 2 Division of Hospital Medicine, University of North Carolina at Chapel Hill, 101 Manning Drive, CB #7085, Chapel Hill, NC 27599 USA

**Keywords:** Verbal autopsy, Routine data, Civil registration, Vital statistics, Global burden of disease

## Abstract

Please see related article: http://bmcmedicine.biomedcentral.com/articles/10.1186/s12916-020-01520-1.

## Background

Well-functioning civil registration and vital statistics (CRVS) systems provide policymakers and stakeholders with accurate and timely information regarding the number of births, deaths, and specific causes of mortality within a population. Ideally, this information is used to guide the effective delivery of health and social development programs [1]. These systems can also facilitate efforts to promote equity and justice by demonstrating the needs of the poorest and most vulnerable groups.

However, many low and middle-income countries (LMICs) – those with the greatest potential benefit from such information – depend on facility-based systems that generate data only on the subset of the population that reaches a health facility [2]. In Uganda, for example, recent national surveys suggest that less than one-third of children under 5 years of age, and less than one-quarter of deaths, are registered with civil authorities, with the lowest rates seen among poor and rural respondents. Failing to capture these vital events creates an “invisible population,” which exacerbates already stark divides between rich and poor, urban and rural [3].

While civil registration with high and representative coverage remains the long-term goal, investment in complementary approaches is urgently needed. This will not only accelerate the design, implementation, and evaluation of cost-effective interventions, but also help to achieve the Sustainable Development Goal (SDG) of 100% birth and 80% death registration [2, 4, 5]. Verbal autopsies (VA), which involve interviews with next of kin or caregivers of the deceased, have been adopted as a practical means of determining the cause of death in areas where CRVS systems are weak [6, 7]. While imperfect, there is ample evidence that VA-based algorithms yield cause of death estimates comparable to other methods [8, 9].

## How good is “good enough?”

The article by Hazard et al. [10] describes the implementation and scale-up of SmartVA, an automated verbal autopsy data collection tool, across multiple countries and contexts. The effort required to roll out this ambitious program, which is supported by Bloomberg Philanthropies’ Data for Health (D4H) Initiative, represents a sizeable achievement in itself and we should not overlook its potential impact on long-term public health.

The authors report estimated cause-specific mortality fractions (CSMF) derived from more than 55,000 events, occurring in four countries analyzed using the SmartVA-Analyze tool. Myanmar and Bangladesh account for about 90% of deaths. It should be highlighted that most of these events would have not otherwise been captured by existing CRVS systems. Had these deaths been documented, many would have not been assigned a cause of death, let alone an accurate one. These results thus represent a major step towards achieving both the SDG targets, as well as more equitable representation in routine data.

Operational successes aside, these findings raise some important questions. For example, what is the appropriate comparator, if any, that we should be using to validate and monitor VA-based CRVS systems? Undoubtedly, the data produced by VA is better than that which existed prior to implementation (i.e. no data), but is this good enough? In the short-term, the answer is probably “yes.” For many areas of the world, simply having access to rigorously collected and systematically analyzed data, even if imperfect, may accelerate the delivery of needed services, advance the effective allocation of limited resources, and help to engage poor and rural communities in policymaking. Over the longer term, however, the answer is less clear and highly context-dependent. VA-based interventions are still considered a stop-gap measure, filling a void until more rigorous, traditional CRVS systems are established. Identifying the time points at which transition from VA-based to traditional CRVS systems should occur will be a key challenge.

In the interim, what should be done when VA analysis is unable to determine a cause of death, such as in approximately 18% of events recorded in Myanmar and Pakistan? Here, the SmartVA-Analyze software redistributed these deaths based on demographic covariates and country-specific patterns of mortality, derived from the Global Burden of Disease (GBD) study. This approach likely biases the results towards the comparator, which – in this case – is the very same GBD study. As the authors note, the comparison was not intended for validation, but rather as a convenient (and perhaps the only available) measure of plausibility.

Should we, however, redistribute deaths at all? While the GBD study data probably represents the best available data to inform redistribution, its uncertainty is likely to be greatest in the places where VA-based CRVS systems are most needed. Notably, in Papua New Guinea and the Philippines, there were fewer deaths with unknown cause (6 and 5%, respectively), and the results of the VA analysis differed in important ways from the GBD estimates. Therefore, are we doing a disservice to poor and rural populations by assigning attribution to their deaths using data that is more likely to be derived from populations with more resources and better access to care? Is this just another form of invisibility, albeit occurring in a much smaller proportion of deaths than before? Ideally, deaths of underdetermined cause should prompt further investigation, especially if clustered in space or time. These deaths might be prioritized for physician review, as was done in the Philippines, as a means of more effectively applying limited resources.

## Conclusions

The findings presented in this article provide further evidence that implementation of VA-based tools to augment CRVS systems in LMICs is feasible, acceptable, and effective. Context-specific experience should guide iterative development and refinement of data collection software, protocols, and analysis algorithms, with particular attention being paid to addressing unknown causes of death. However, this should not delay the implementation of an evidence-based strategy to overcome a critical, yet largely neglected issue of social justice and public health.

## Data Availability

Not applicable.
